# E-Sports Training System Based on Intelligent Gesture Recognition

**DOI:** 10.1155/2022/2689949

**Published:** 2022-07-09

**Authors:** Hui Li, Yao Lu, Hongqiao Yan

**Affiliations:** School of Sport Communication and Information Technology, Shandong Sport University, Jinan 250000, China

## Abstract

In order to improve the effect of e-sports training, this paper combines the intelligent gesture recognition technology to construct an e-sports training system and judges the training effect of players through the recognition of players' gestures. Moreover, this paper studies the commonly used feature extraction algorithms and proposes an improved SLC-Harris feature extraction algorithm, and the feasibility of this algorithm is verified by the experimental results on the EUROC data set. In addition, this paper uses the KLT optical flow algorithm to track the extracted feature points and calculates the pure visual pose through epipolar geometry, triangulation, and PnP algorithms. The experimental research results show that the electronic economic training system based on intelligent gesture recognition proposed in this paper has certain effects.

## 1. Introduction

The reason why e-sports can become a sports competition is that it is closely related to the progress of society, the development of science and technology, and the spiritual and cultural needs of the people. Although there are countless people who enjoy this high-tech intellectual sports event, in fact, public opinion instills the harmful opinion of e-sports in people intentionally or unintentionally. Some media reported extensively that some students were addicted to games and could not extricate themselves, wasting their youth and studies, which made e-sports become “electronic heroin” that everyone shouted. The huge pressure of public opinion makes e-sports face severe survival pressure, and it is difficult for enterprises to enter this market justifiably. Moreover, athletes can only be called “players,” and their treatment cannot be compared with that of ordinary athletes. At the same time, the majority of fans can only engage in e-sports secretly. In addition, in the face of huge pressure from public opinion, it is difficult for the government to guide and supervise confidently, and sometimes, it has to prohibit escrow. The ban on television broadcasting of e-sports competitions can be described as a huge obstacle to the normal development of the current social discrimination against the e-sports sports industry.

Generally speaking, the development of e-sports is not yet mature, and the development of e-sports is still in its infancy [1], which is manifested in many aspects: the public recognition is not enough, there are few related large-scale events, there is no professional-scale operation, there is less research in this area, and so on [2]. Especially on college campuses, although students have more time for self-discipline than before, the school does not pay enough attention to e-sports, and there is no relatively formal organization and management of participants, which has led to many human resources problems waste [3].

In order to cater to the trend of e-sports development, vigorously develop e-sports business, improve the overall level of e-sports, and enable e-sports activities to develop well in colleges and universities, the current primary task is to deepen the characteristics of students participating in e-sports activities [4]. Among them, the analysis and research on the current situation, development trend, and participation significance of e-sports in colleges and universities are particularly important in order to discover the problems existing in the development of campus e-sports and put forward reasonable suggestions for the development [5].

As emerging sports, e-sports are mainly participated by the younger generation, which has the characteristics of younger and younger age. E-sports can exercise people's thinking ability, psychological pressure resistance, unity and cooperation, hand-eye coordination, and so on. It can also make the younger generation have the awareness of abiding by the rules in the process of participating in e-sports [6]; trained participants have a fair and open, never admit defeat, pursue a stronger competitive spirit, and have a positive impact on the lives of participants. Many colleges and universities have successively opened related majors in e-sports. Although e-sports is popular in the world, the related research and guiding theories on how to cultivate e-sports talents are rare [7].

Different scholars have different views on the attributes and characteristics of e-sports. Literature [8] proposed that “e-sports include three basic characteristics: one is electronics, the other is competitive sports, and the third is a confrontation between people. At the same time, e-sports sports are divided into virtualized e-sports sports and fictionalized sports.” Literature [9] pointed out that “the most fundamental characteristics of video games that distinguish them from other artificial games are: virtual environment, absence of the body and artificial intelligence,” emphasizing the main position of electronic communication technology in e-sports. Scholar Yang Fang believes that “e-sports should return to the essence of games, and games to competitive sports are based on the evolution trend of play-game-competitive sports” and based on the development process of traditional competitive sports, puts forward a plan for the development of e-sports. Jia Peng and Yao Jiaxin believe that e-sports has great characteristics: the diversity of functional structure requirements, the full expansion of self-awareness, the complexity of sports information pattern recognition, the agility of information processing efficiency, and the accuracy of intuitive thinking and decision-making. Sex analyzes and clarifies the various attributes of e-sports from many aspects [10].

The discussion on the attributes of e-sports is still going on. Based on the current research, it can be determined that the two essential attributes of e-sports are electronic interaction and confrontational competition. Without electronic interaction, it becomes traditional competitive sports; it becomes a video game, so the two are interdependent and indispensable. With the development of electronic interaction technology, various forms of e-sports have emerged [11].

Event services are mainly engaged in e-sports referees, coaches, club operation and management, game commentary, data and tactical analysis, and so on. Practitioners need to have data analysis capabilities, management capabilities, and commentary capabilities. The production and broadcast of the event include content production and external dissemination, mainly involving the design of live content and promotion plans, venue layout, equipment debugging, video data collection, postprocessing, background data analysis, and so on. The practitioners should have journalism, communication, broadcasting, TV technology, and other related professional abilities [12].

Since the e-sports industry is an emerging industry, most employees are not from e-sports majors and have not received a complete and systematic e-sports theoretical education, but nearly 90% of the employees believe that the e-sports industry needs prejob training [13]. Judging from the current situation of the development of the entire industry, it is undoubtedly the most attractive option to work for game manufacturers, but it is difficult for game manufacturers to absorb more human resources without major business adjustments. Therefore, the need to train practitioners in support organizations around e-sports events becomes more obvious [14]. For example, training content production capabilities (reporters, screenwriters, copywriters, and anchors) requires a professional background in journalism and communication; training event support capabilities (coaches, data analysts, nutritionists, and brokers) requires sports and information technology. Professional background: training public relations, marketing capabilities (products, business, brand marketing, and media), requires a professional background in marketing and management [15].

E-sports self-media is still a media, and you must have the ability to report news, or you can dig deep into a vertical field, such as specializing in video commentary of games, specializing in game clearance strategies, specializing in sharing game skills, and so on. After all, hot spots can bring traffic. WeMedia is a personalized media with social attributes; it communicates with users; and it has its own distinct character orientation [16]. To be a self-media, you should also have strong analytical skills and be able to interpret a topic or special event from a unique or professional perspective. Current e-sports professional ability training pathways.

### 1.1. Current E-Sports Professional Ability Training Pathways

Most training institutions in society position themselves as training professional players but basically lack training resources. Training institutions do not have coaches, data analysts, or club managers, and it is difficult for the trained people to find a suitable position in the e-sports circle. Rather than cultivating professional skills, it is better to make money from e-sports hot spots. Money has no intention or inability to contribute to the development of the e-sports industry [17].

At present, the main e-sports talents are cultivated by e-sports companies and e-sports clubs. The club mainly trains professional players, coaches, and data analysts in order to achieve better results in the league. Game companies train referees, game developers, commentators, and other related talents to ensure the healthy development of the e-sports industry [18]. An analysis of the revenue structure of the e-sports industry can help us see the e-sports industry more transparently. The truly profitable institutions are still game manufacturers, which continuously create market value through development and operation. In the context of the continuous development and popularization of the video game industry, competition has become a starting point for expanding influence and creating new commercial value. The comprehensive development of competitive value is inseparable from the promotion of surrounding formats, and new jobs such as video, live broadcast, and commentary emerge in an endless stream [19].

This paper combines the intelligent gesture recognition technology to construct an e-sports training system and uses the player's gesture recognition to judge the player's training effect to improve the e-sports training effect.

## 2. Intelligent Gesture Recognition

### 2.1. Gesture Intelligent Positioning

**Figure 1:**
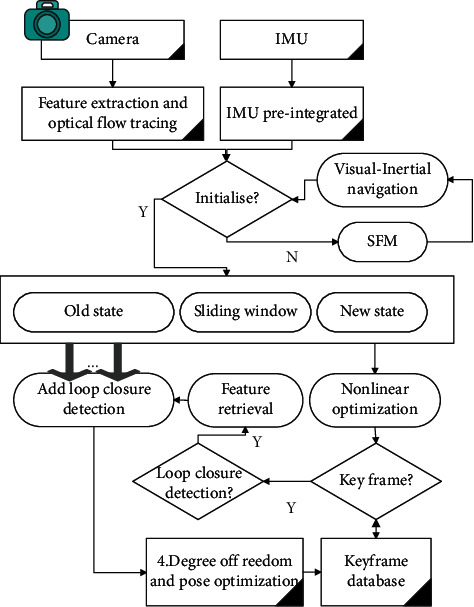
Gesture positioning algorithm structural framework.

The structural framework of the gesture autonomous localization algorithm is shown in Figure 1.

**Figure 2:**
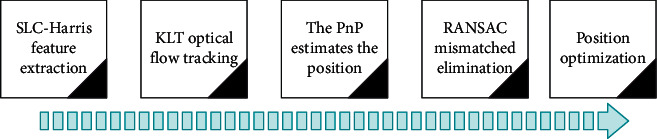
Front-end flowchart.

Monocular visual-inertial odometry uses a pure camera in the front end for motion estimation. The algorithm firstly extracts the features of the image information collected by the camera, then uses the optical flow method to track the feature points, and finally uses PnP (Perspective-n-Point) to perform motion estimation on the tracked feature points. Then, the algorithm eliminates the mismatched point pairs through random sampling consistency (RANSAC) and uses nonlinear optimization to optimize the pose. The front-end process is shown in Figure 2.

### 2.2. SLC-Harris Feature Extraction

The feature is the digital expression of the object in the image, and the image can be quantitatively analyzed by extracting the feature. Commonly used feature extraction methods mainly include SIFT algorithm, SURF algorithm, and ORB algorithm.

The traditional Harris algorithm calculates the angular responsivity as shown below. It is mainly based on the weighted summation of the squared and multiplied gradients of all pixels in the window.

(1) $$\begin{array}{c} R=\left|C\right|-k\times {\left(\text{trace}\left(C\right)\right)}^{2}. \end{array}$$

Among them, there are

(2) $$\begin{array}{c} \left|C\right|=\lambda_{1}\times \lambda_{2}, \end{array}$$

(3) $$\begin{array}{c} \text{trace}\left(C\right)=\lambda_{1}+\lambda_{2}. \end{array}$$

In formula (1), *k* is a constant ranging from 0.04 to 0.06, and both *λ*_1_ and *λ*_2_ in formula (2) represent eigenvalues.

**Figure 3:**
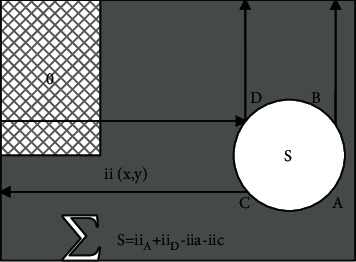
Rectangular window pixel calculation.

For a grayscale image, the value of any point (*x*, *y*) in the integral image ii (*x*, *y*) refers to the sum of all grayscale values from the upper left corner of the image to the area where this point is located, as shown in Figure 3.

The calculation formula of pixels in the rectangular window is as follows:

(4) $$\begin{array}{c} ii\left(x,y\right)=\sum_{x^{\prime }\leq x,y^{\prime }\leq y}I\left(x^{\prime },y^{\prime }\right). \end{array}$$

The most complex calculation in the Harris algorithm is the calculation of diagonal responsivity. The original calculation method causes the calculation overlap between each pixel in the integration window, resulting in high computational complexity. For this, the gradient values in *g*_*x*_^2^, *g*_*y*_^2^ and *g*_*x*_*g*_*y*_ are used to integrate the image to speed up the calculation of the angular responsivity. The calculation formula is as follows:

(5) $$\begin{array}{c} ii_{xx}\left(x,y\right)=\sum_{x^{\prime }\leq x,y^{\prime }\leq y}g_{x}^{2}\left(x^{\prime },y^{\prime }\right),\\ ii_{yy}\left(x,y\right)=\sum_{x^{\prime }\leq x,y^{\prime }\leq y}g_{y}^{2}\left(x^{\prime },y^{\prime }\right),\\ ii_{xy}\left(x,y\right)=\sum_{x^{\prime }\leq x,y^{\prime }\leq y}g_{x}\left(x^{\prime },y^{\prime }\right)g_{y}\left(x^{\prime },y^{\prime }\right). \end{array}$$

**Figure 4:**
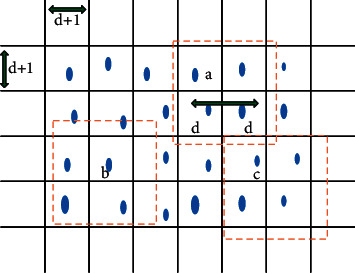
Efficient nonmaximum suppression.

Efficient nonmaximum suppression (E-NMS) is used to efficiently extract unique feature locations for each corner region, and the region thresholds are compared using image patches instead of pixels. The principle is shown in Figure 4.

### 2.3. KLT Optical Flow Tracking

After the key points are extracted, the optical flow method is used to calculate the minimum photometric error by establishing an error model. This method does not need to calculate descriptors or feature point matching, which will greatly save the amount of calculation.

The basic idea of LK optical flow is to assume that the optical flow in the local neighborhood of a pixel is invariant, and based on this assumption, construct a least-squares problem about the optical flow of the neighborhood pixels.

First, it is assumed that the light intensity of the pixel in each frame of the image is constant. According to this, for the pixel located at (*x*, *y*) at time *t*, moving to (*x* + *dx*, *y* + *dy*) at time *t* + *dt*, there are

(6) $$\begin{array}{c} I\left(x,y,t\right)=I\left(x+dx\;,y+dy\;,t+dt\right). \end{array}$$

Then, according to another basic assumption of LK optical flow, the displacement of pixels in adjacent images is small; the Taylor expansion of formula (6) is

(7) $$\begin{array}{c} I\left(x+dx\;,y+dy\;,t+dt\right)\approx I\left(x,y,t\right)+\frac{\partial I}{\partial x}dx+\frac{\partial I}{\partial y}dy+\frac{\partial I}{\partial t}dt. \end{array}$$

Combining the above formulas and dividing by *dt* into both sides of the formula, we get:

(8) $$\begin{array}{c} \frac{\partial I}{\partial x}\frac{\text{d}x}{\text{d}t}+\frac{\partial I}{\partial y}\frac{\text{d}y}{\text{d}t}=-\frac{\partial I}{\partial t}, \end{array}$$

where d*x*/d*t* represents the motion speed of the pixel on the *x*-axis, d*y*/d*t* represents the motion speed of the pixel on the *y*-axis, and the two speeds are recorded as *u* and *v*, respectively. At the same time, *∂I*/*∂x* represents the gradient value of the image in the *x*-axis direction at the pixel point; *∂I*/*∂y* represents the gradient value in the *y*-axis direction at the pixel point; and *∂I*/*∂t* represents the derivative value of the image in the *t* direction, which are denoted as *I*_*x*_, *I*_*y*_, and *I*_*t*_, respectively. Therefore, formula (8) can be written in matrix form as follows:

(9) $$\begin{array}{c} \left[\begin{array}{cc} I_{x} & I_{y} \end{array}\right]\left[\begin{array}{c} u\\ v \end{array}\right]=-I_{t}. \end{array}$$

Finally, according to the third basic assumption of the LK optical flow method, adjacent pixels in the same image plane have similar motion; a *w* × *w* size window is defined. According to the same motion of all pixels in the window, *w*^2^ formulas can be listed; the overdetermined formulas can be constructed; and the motion parameters of the center point can be obtained by the least square method. Accordingly, its formula can be expressed as follows:

(10) $$\begin{array}{c} \left(\begin{array}{cc} \sum I_{x}I_{x} & \sum I_{x}I_{y}\\ \sum I_{x}I_{y} & \sum I_{y}I_{y} \end{array}\right)\left(\begin{array}{c} u\\ v \end{array}\right)=-\left(\begin{array}{c} \sum I_{x}I_{t}\\ \sum I_{y}I_{t} \end{array}\right). \end{array}$$

Each image frame is downsampled by pyramid layering, and multilevel pyramids are established.

(11) $$\begin{array}{c} I^{L}\left(x,y\right)=\frac{1}{4}I^{L-1}\left(2x,2y\right)+\frac{1}{8}\left(\begin{array}{c} I^{L-1}\left(2x-1,2y\right)+I^{L-1}\left(2x+1,2y\right)\\ +I^{L-1}\left(2x,2y-1\right)+I^{L-1}\left(2x,2y+1\right) \end{array}\right)\\ +\frac{1}{16}\left(\begin{array}{c} I^{L-1}\left(2x+1,2y+1\right)+I^{L-1}\left(2x+1,2y+1\right)+\\ I^{L-1}\left(2x+1,2y+1\right)+I^{L-1}\left(2x+1,2y+1\right) \end{array}\right), \end{array}$$

where *L* represents the *L*th layer image.

The algorithm calculates the value of the bottom layer from top to bottom according to the Gaussian pyramid and calculates the pixel value near the edge of the image based on the following formulas:

(12) $$\begin{array}{c} I^{L-1}\left(-1,y\right)\approx I^{L-1}\left(-1,y\right),\\ I^{L-1}\left(x,-1\right)\approx I^{L-1}\left(x,0\right),\\ I^{L-1}\left(n_{x}^{L-1},y\right)\approx I^{L-1}\left(n_{x}^{L-1}-1,y\right),\\ I^{L-1}\left(x,n_{y}^{L-1}\right)\approx I^{L-1}\left(x,n_{y}^{L-1}-1\right),\\ I^{L-1}\left(n_{x}^{L-1},n_{y}^{L-1}\right)\approx I^{L-1}\left(n_{x}^{L-1}-1,n_{y}^{L-1}-1\right). \end{array}$$

**Figure 5:**
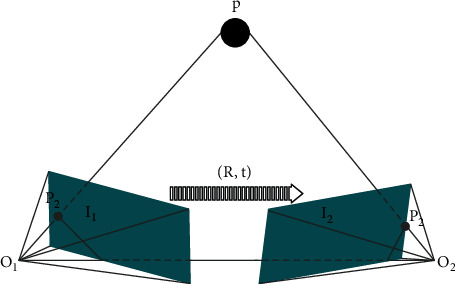
Epipolar geometric constraints.

The camera motion pose is estimated using SFM in the vision front end. For a monocular camera, the camera pose can be estimated by the geometric relationship between two points in different locations in real space and their projected points on their respective imaging planes. As shown in Figure 5, *P* is any point in the three-dimensional space, and its coordinates are [*X*, *Y*, *Z*]^*T*^; *O*_1_ and *O*_2_ are the optical centers of the two camera positions. *p*_1_ and *p*_2_ are the projection points of point P on the imaging plane *I*_1_ and the imaging plane *I*_2_, respectively. According to the pixel positions of the two matched point pairs *p*_1_ and *p*_2_, the essential matrix *E* and the fundamental matrix *F* can be obtained.

According to the camera imaging model, we assume that *K* is the camera internal parameter matrix, and *R* and *t* represent the rotation matrix and translation vector from plane *I*_1_ to plane *I*_2_, and the following formula can be obtained:

(13) $$\begin{array}{c} s_{1}p_{1}=KP,s_{2}p_{2}=K\left(RP+t\right). \end{array}$$

Homogeneous coordinate transformation and normalization between 2D and 3D, we can get

(14) $$\begin{array}{c} x_{1}=K^{-1}p_{1},x_{2}=K^{-1}p_{2}, \end{array}$$

where *x*_1_ and *x*_2_ represent the coordinates of pixels *p*_1_ and *p*_2_ in the normalized plane, respectively. The algorithm combines formuls (13) and (14) and multiplies by *x*_2_^*T*^*t*^∧^ to obtain the essential matrix *E* and the fundamental matrix *F*, which can be sorted out as follows:

(15) $$\begin{array}{c} p_{2}^{T}K^{-T}t^{\wedge }RK^{-1}p_{1}=0,\\ E=t^{\wedge }R,F=K^{-T}EK^{-1}, \end{array}$$

where *t*^∧^ represents the antisymmetric matrix.

When there are more than eight sets of point pairs such as *p*_1_ and *p*_2_, the eight-point method can be used to construct a linear formula system for the simplified formula, and then the unique solution of *R* and *t* can be obtained.

(16) $$\begin{array}{c} x_{2}^{T}Ex_{1}=p_{2}^{T}Fp_{1}=0. \end{array}$$

When the monocular camera recovers the pose through the epipolar geometric relationship, the obtained translation is the normalized value, which has no practical significance. In order to obtain the depth information on feature points, triangulation needs to be introduced. We assume that *s*_1_ and *s*_2_ represent the depth of the two feature points; we can get

(17) $$\begin{array}{c} s_{2}x_{2}=s_{1}Rx_{1}+t. \end{array}$$

The feature point depth values *x*_2_^∧^ and *x*_1_∧ can be obtained by left-multiplying formula (17) by *s*_1_ or *s*_2_, respectively, as follows:

(18) $$\begin{array}{c} s_{1}x_{2}^{\wedge }Rx_{1}+x_{2}^{\wedge }t=0,\\ s_{2}x_{1}^{\wedge }Rx_{2}-x_{1}^{\wedge }t=0. \end{array}$$

**Figure 6:**
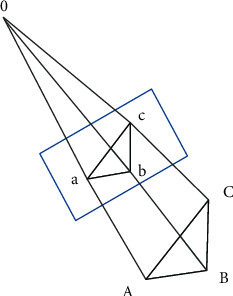
Schematic diagram of P3P relationship.

When the positions of multiple points in space are known, the camera pose can be estimated by the PnP algorithm. Common PnP algorithms include P3P, DLT, and BA optimization. Among them, the P3P algorithm is the most common method. The algorithm needs to know at least three points and their projection points on the camera imaging plane. Then, the camera pose can be estimated by solving the relationship between point pairs according to the similar triangle principle and the cosine theorem. A schematic diagram of the P3P relationship is shown in Figure 6.

**Figure 7:**
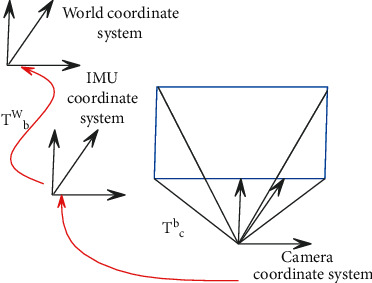
Coordinate conversion relationship.

The coordinate system convention is as follows: the world coordinate system is represented by (·)^*w*^, and (·)^*b*^ and (·)^*c*^ represent the IMU coordinate system and the camera coordinate system, respectively. The relationship between the coordinate systems is shown in Figure 7. (.)^*v*^ represents the visual reference frame in the sliding window, which is independent of the IMU measurement and can represent any frame in the visual structure. (.)_*b*_^*w*^ represents the transformation from the IMU coordinate system to the world coordinate system; *b*_*k*_ represents the IMU frame of the *k*th image; (·)_*c*_^*v*^ represents the transformation from the camera coordinate system to the visual reference system; and *c*_*k*_ represents the camera frame of the *k*th image. $\hat{\left(\cdot \right)}$ represents the measured value and parameter estimation value of the sensor; $\bar{\left(\cdot \right)}$ represents the latest scale parameter of the sliding window; and the rotation can be represented by the rotation matrix **R** and the quaternion **q**. **g**^*w*^=[0,0, *g*]^*T*^ represents the gravity vector in the world coordinate system, and *g*^*v*^ represents the gravity vector in the visual reference coordinate system.

### 2.4. IMU Preintegration

**Figure 8:**
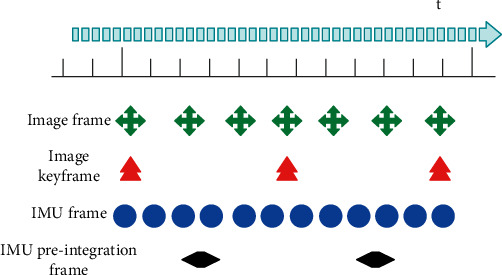
Principle of preintegration.

The sampling frequency of the camera used in this paper is 20 Hz, and the sampling frequency of the IMU is 200 Hz. It can be seen that the frequency of the IMU is much higher than that of the image. In order to avoid the repeated integration phenomenon caused by the frequency change of the visual frame optimization state caused by the high sampling rate of the IMU, a preintegration technique is used for all IMU sampling data between two image key frames. Furthermore, inertial measurements between adjacent image key frames are aggregated into a relative motion constraint through a preintegration technique. The principle of preintegration is shown in Figure 8.

In Figure 8, from top to bottom are the time scale line, the number of image frames generated, the number of image key frames generated, the number of IMU samples, and the IMU preintegration value.

The measurement error of the system is mainly affected by bias random walk *b* and white noise *η*, and other errors such as the Markov process are ignored. Then, the measurement model of the accelerometer and gyroscope in the IMU can be expressed by the following formula:

(19) $$\begin{array}{c} {\hat{\omega }}^{b}=\omega^{b}\left(t\right)+b_{\omega }+\eta_{\omega }\frac{1}{2},\\ {\hat{a}}^{b}=q_{b}^{w^{T}}\left(a^{w}-g^{w}\right)+b_{a}+\eta_{a}, \end{array}$$

where ${\hat{\omega }}^{b},{\hat{a}}^{b}\left(t\right),\omega^{b}\left(t\right)$, and *a*^*w*^(*t*) represent the measured value and real value of angular velocity and acceleration, respectively; *b*_*ω*_, *b*_*a*_, *η*_*ω*_, and *η*_*a*_ represent the random walk noise and measurement white noise of angular velocity and acceleration, respectively; and *q*_*b*_^*w*^*T*^^ is the rotation matrix transformed from the world coordinate system to the IMU coordinate system.

White noise obeys a Gaussian distribution, that is, *η*_*a*_ ~ *N*(0, *σ*_*a*_^2^), *η*_*ω*_ ~ *N*(0, *σ*_*ω*_^2^). The derivative of random walk noise also obeys the Gaussian distribution, that is, *η*_*b*_*a*__ ~ *N*(0, *σ*_*b*_*a*__^2^), *η*_*b*_*ω*__ ~ *N*(0, *σ*_*b*_*ω*__^2^).

The differential kinematic formulas for *P*, *V*, *Q* (representing the position, velocity, and rotation expressed in quaternions, respectively) versus time can be written as follows:

(20) $$\begin{array}{c} {\dot{p}}_{b_{t}}^{w}=v_{t}^{w},\\ {\dot{v}}_{t}^{w}=a_{t}^{w},\\ {\dot{q}}_{b_{t}}^{w}=q_{b_{t}}^{w}⊗\left[\begin{array}{c} 0\\ \frac{1}{2}\omega^{b_{t}} \end{array}\right], \end{array}$$

where ⊗ represents quaternion multiplication.

Through the above derivative relationship, the position, velocity, and rotation at time *k* + 1 can be obtained from the position, velocity, and rotation at time *k* and by integrating the measured values of the IMU over time Δ*t*_*k*_. The continuous integration formula for PVQ is as follows:

(21) $$\begin{array}{c} p_{b_{k+1}}^{w}=p_{b_{k}}^{w}+v_{b_{k}}^{w}\Delta t_{k}+\int \int_{t\in \left[k,k+1\right]}\left[R_{t}^{w}\left({\hat{a}}_{t}-b_{a_{t}}\right)-g^{w}\right]\text{d}t^{2}\text{,}\\ v_{b_{k+1}}^{w}=v_{b_{k}}^{w}+\int_{t\in \left[k,k+1\right]}\left[R_{t}^{w}\left({\hat{a}}_{t}-b_{a_{t}}\right)-g^{w}\right]\text{d}t,\\ q_{b_{k+1}}^{w}=q_{b_{k}}^{w}⊗\int_{t\in \left[k,k+1\right]1/2}\Omega \left({\hat{\omega }}_{t}-b_{w_{t}}\right)q_{t}^{b_{k}}\text{d}t, \end{array}$$

where ${\hat{a}}_{t}$ and ${\hat{\omega }}_{t}$ represent the acceleration and angular velocity measured in the IMU coordinate system, respectively. Δ*t*_*k*_ represents the time difference from the *k*th frame to the *k* + 1 frame. *R*_*t*_^*w*^ represents the rotation matrix from the world coordinate system to the IMU coordinate system. Because the measured ${\hat{a}}_{t}$ and ${\hat{\omega }}_{t}$ belong to the IMU coordinate system, in order to transform the IMU measured value to the world coordinate system, the rotation matrix needs to be left-multiplied. Ω(*ω*) means quaternion right multiplication; *ω*_*x*_ means antisymmetric matrix in quaternion multiplication (*ω* means the imaginary part value of quaternion). We assume that the quaternion is $q=\left[\begin{array}{cccc} x & y & z & s \end{array}\right]=\left[\begin{array}{cc} \omega & s \end{array}\right]$; then we have

(22) $$\begin{array}{c} \Omega \left(\omega \right)=\left[\begin{array}{cc} -\omega_{\times } & \omega \\ -\omega^{T} & 0 \end{array}\right],\\ \omega_{\times }=\left[\begin{array}{ccc} 0 & \omega_{z} & \omega_{y}\\ \omega_{z} & 0 & -\omega_{x}\\ -\omega_{y} & \omega_{x} & 0 \end{array}\right]. \end{array}$$

By observing the continuous integral formula of PVQ, it can be seen that the current state is recursively obtained from the state of the previous time, and the estimated value is constantly changing. This will cause the IMU measurements to be repropagated, causing the velocity and rotation to be reintegrated after each nonlinear optimization iteration, resulting in a higher computational cost. Therefore, the optimization variables are separated from the IMU preintegration terms of the two key frames, and the rotation matrix *R*_*w*_^*b*_*k*_^ of the world coordinate system to the IMU coordinate system can be obtained by simultaneously left-multiplying the left and right sides of the continuous integration formula of PVQ:

(23) $$\begin{array}{c} R_{w}^{b_{k}}p_{b_{k+1}}^{w}=R_{w}^{b_{k}}\left(p_{b_{k}}^{w}+v_{b_{k}}^{w}\Delta t_{k}-\frac{1}{2}g^{w}\Delta t_{k}^{2}\right)+\alpha_{b_{k+1}}^{b_{k}},\\ R_{w}^{b_{k}}v_{b_{k+1}}^{w}=R_{w}^{b_{k}}\left(v_{b_{k}}^{w}-g^{w}\right)+\beta_{b_{k+1}}^{b_{k}},\\ q_{w}^{b_{k}}⊗q_{b_{k+1}}^{w}=\gamma_{b_{k+1}}^{b_{k}}. \end{array}$$

The image frames *b*_*k*_ and *b*_*k*+1_ of two consecutive moments are given, and the linear acceleration and angular velocity are preintegrated in the local coordinate system *b*_*k*_ to obtain

(24) $$\begin{array}{c} \alpha_{b_{k+1}}^{b_{k}}=\int \int_{t\in \left[k,k+1\right]}R_{t}^{b_{k}}\left({\hat{a}}_{t}-b_{a_{t}}\right)\text{d}t^{2},\\ \beta_{b_{k+1}}^{b_{k}}=\int_{t\in \left[k,k+1\right]}\left[R_{t}^{b_{k}}\left({\hat{a}}_{t}-b_{a_{t}}\right)\right]\text{d}t,\\ \gamma_{b_{k+1}}^{b_{k}}=\int_{t\in \left[k,k+1\right]2}\left(1/2\right)\Omega \left({\hat{\omega }}_{t}-b_{\omega_{t}}\right)\gamma_{t}^{b_{k}}\text{d}t, \end{array}$$

where *α*_*b*_*k*+1__^*b*_*k*_^, *β*_*b*_*k*+1__^*b*_*k*_^, *γ*_*b*_*k*+1__^*b*_*k*_^ represent the relative pose, velocity, and rotation constraints, respectively, and are also the relative motion of *b*_*k*+1_ to *b*_*k*_. It can be seen that they are only related to ${\hat{a}}_{t}$ and ${\hat{\omega }}_{t}$ in *b*_*k*_ and *b*_*k*+1_, and they have nothing to do with the initial position and velocity of coordinate system *b*_*k*_.

Therefore, the preintegration formula is rediscussed, in terms of *α*_*b*_*k*+1__^*b*_*k*_^; it is related to ${\hat{a}}_{t}$ and ${\hat{\omega }}_{t}$ of the IMU; and ${\hat{a}}_{t}$ and ${\hat{\omega }}_{t}$ are also variables that need to be optimized. When the bias change is small, *α*_*b*_*k*+1__^*b*_*k*_^, *β*_*b*_*k*+1__^*b*_*k*_^, *γ*_*b*_*k*+1__^*b*_*k*_^ are adjusted according to their first-order approximations to the bias.

(25) $$\begin{array}{c} \alpha_{b_{k+1}}^{b_{k}}\approx {\hat{\alpha }}_{b_{k+1}}^{b_{k}}+J_{b_{a}}^{\alpha }\delta b_{a}+J_{b_{\omega }}^{\alpha }\delta b_{\omega },\\ \beta_{b_{k+1}}^{b_{k}}\approx {\hat{\beta }}_{b_{k+1}}^{b_{k}}+J_{b_{a}}^{\beta }\delta b_{a}+J_{b_{\omega }}^{\beta }\delta b_{\omega },\\ \gamma_{b_{k+1}}^{b_{k}}\approx \gamma_{b_{k+1}}^{b_{k}}⊗\left[\begin{array}{c} 0\\ \frac{1}{2}J_{b_{a}}^{\gamma }\delta b_{\omega } \end{array}\right], \end{array}$$

where *J*_*b*_*a*__^*α*^ and *J*_*b*_*ω*__^*α*^ are the block matrices in *J*_*b*_*k*+1__^*α*^ and *J*_*b*_*a*__^*β*^ and *J*_*b*_*ω*__^*β*^ are the block matrices in *J*_*b*_*k*+1__^*β*^.

There are errors in the integral values of the IMU at different times, and the errors at time *t* are mainly related to the measured values of *α*_*t*_^*b*_*k*_^, *β*_*t*_^*b*_*k*_^, *θ*_*t*_^*b*_*k*_^, *b*_*a*_*t*__, and *b*_*w*_*t*__ at time *t*. The following definition is given to represent the error vector:

(26) $$\begin{array}{c} \delta z_{t}^{b_{k}}=\left[\begin{array}{c} \delta \alpha_{t}^{b_{k}}\\ \delta \beta_{t}^{b_{k}}\\ \delta \theta_{t}^{b_{k}}\\ \delta b_{a_{t}}\\ \delta b_{\omega_{t}} \end{array}\right]. \end{array}$$

The derivation is based on the derivative of the error term kinetic formula. First, two concepts are introduced: true and nominal, where true represents the real measurement value containing noise and nominal represents the theoretical value without noise, and *δ* represents the measurement error; there are

(27) $$\begin{array}{c} \delta \dot{\alpha }={\dot{\alpha }}_{\text{true}}-{\dot{\alpha }}_{\text{nominal}},\\ \delta \dot{\beta }={\dot{\beta }}_{\text{true}}-{\dot{\beta }}_{\text{nominal}}. \end{array}$$

Among them, there are:

(28) $$\begin{array}{c} {\dot{\beta }}_{\text{true}}=R_{t}^{b_{k}}\left[{\hat{a}}_{t}-\eta_{a}-b_{a_{t}}-\delta b_{a_{t}}-\left[{{\hat{a}}_{t}-b_{a_{t}}}_{\times }\delta \theta \right.\right],\\ {\dot{\beta }}_{\text{nominal}}=R_{t}^{b_{k}}\left({\hat{a}}_{t}-b_{a_{t}}\right). \end{array}$$

Combining the above formulas, we can obtain

(29) $$\begin{array}{c} \delta \dot{\beta }=-R_{t}^{b_{k}}{{\hat{a}}_{t}-b_{a_{t}}}_{\times }\delta \theta -R_{t}^{b_{k}}\delta b_{a_{t}}-R_{t}^{b_{k}}\eta_{a}. \end{array}$$

The derivation of $\delta \dot{\theta }$ is as follows, and according to the formula in the literature, it can be known that

(30) $$\begin{array}{c} q_{\text{true}}=q_{\text{nominal}}⊗\delta q. \end{array}$$

In this paper, according to the noise model and bias, we can get

(31) $$\begin{array}{c} \delta \dot{\theta }\approx -{{\hat{\omega }}_{t}-b_{\omega_{t}}}_{\times }\delta \theta -\eta_{\omega }-\delta b_{\omega_{t}}. \end{array}$$

In summary, the derivative of the IMU measurement error term at time *t* can be as follows:

(32) $$\begin{array}{c} \left[\begin{array}{c} \delta {\dot{\alpha }}_{t}^{b_{k}}\\ \delta {\dot{\beta }}_{t}^{b_{k}}\\ \delta {\dot{\theta }}_{t}^{b_{k}}\\ \delta {\dot{b}}_{a_{t}}\\ \delta {\dot{b}}_{\omega_{t}} \end{array}\right]=\left[\begin{array}{ccccc} 0 & I & 0 & 0 & 0\\ 0 & 0 & -R_{t}^{b_{k}}{{\hat{a}}_{t}-b_{a_{t}}}_{\times } & -R_{t}^{b_{k}} & 0\\ 0 & 0 & -{{\hat{\omega }}_{t}-b_{\omega_{t}}}_{\times } & 0 & -I\\ 0 & 0 & 0 & 0 & 0\\ 0 & 0 & 0 & 0 & 0 \end{array}\right]\left[\begin{array}{c} \delta \alpha_{t}^{b_{k}}\\ \delta \beta_{t}^{b_{k}}\\ \delta \theta_{t}^{b_{k}}\\ \delta b_{a_{t}}\\ \delta b_{\omega_{t}} \end{array}\right]+c\left[\begin{array}{c} \eta_{a}\\ \eta_{\omega }\\ \eta_{b_{a}}\\ \eta_{b_{\omega }} \end{array}\right]. \end{array}$$

We set $F_{t}=\left[\begin{array}{ccccc} 0 & I & 0 & 0 & 0\\ 0 & 0 & -R_{t}^{b_{k}}{{\hat{a}}_{t}-b_{a_{t}}}_{\times } & -R_{t}^{b_{k}} & 0\\ 0 & 0 & -{{\hat{\omega }}_{t}-b_{\omega_{t}}}_{\times } & 0 & -I\\ 0 & 0 & 0 & 0 & 0\\ 0 & 0 & 0 & 0 & 0 \end{array}\right],G_{t}=\left[\begin{array}{cccc} 0 & 0 & 0 & 0\\ -R_{t}^{b_{k}} & 0 & 0 & 0\\ 0 & I & 0 & 0\\ 0 & 0 & I & 0\\ 0 & 0 & 0 & I \end{array}\right]$. The above formula can be simplified to

(33) $$\begin{array}{c} \delta {\dot{z}}_{t}^{b_{k}}=F_{t}\delta z_{t}^{b_{k}}+G_{t}n_{t}. \end{array}$$

According to the definition of the derivative, the prediction formula of the mean is as follows:

(34) $$\begin{array}{c} \delta {\dot{z}}_{t}^{b_{k}}=\underset{\delta_{t}⟶0}{\lim}\frac{\delta z_{t+\delta t}^{b_{k}}-\delta z_{t}^{b_{k}}}{\delta_{t}},\\ \delta z_{t+\delta_{t}}^{b_{k}}=\left(1+F_{t}\delta_{t}\right)\delta z_{t}^{b_{k}}+\left(G_{t}\delta_{t}\right)n_{t}. \end{array}$$

According to the error value at the current moment, the mean and covariance at the next moment can be predicted. The prediction formula for covariance is as follows:

(35) $$\begin{array}{c} P_{t+\delta t}^{b_{k}}=\left(1+F_{t}\delta_{t}\right)P_{t}^{b_{k}}{\left(1+F_{t}\right)}^{T}+\left(G_{t}\delta_{t}\right)Q{\left(G_{t}\delta_{t}\right)}^{T}, \end{array}$$

where *P*_*t*_^*b*_*k*_^ represents the initial value of the iteration and its value is zero and *Q* represents the diagonal covariance matrix of the noise term as follows:

According to formula (35), the iterative formula of the error term Jacobian can be obtained as follows:

(36) $$\begin{array}{c} J_{t+\delta t}=\left(1+F_{t}\right)J_{t}, \end{array}$$

where the iterative initial value of the Jacobian matrix *J*_*t*_ is *I*.

### 2.5. Sliding Window Initialization

When the camera extrinsic parameter matrix $\left({\bar{p}}_{b_{k}}^{v},{\bar{p}}_{c_{k}}^{v}\right)$ is known, the pose obtained by the initialization of the monocular camera is transformed from the visual coordinate system to the IMU coordinate system to obtain the following formula:

(37) $$\begin{array}{c} q_{b_{k}}^{v}=q_{c_{k}}^{v}⊗q_{b}^{v},\\ s{\bar{p}}_{b_{k}}^{v}+q_{c_{k}}^{v}p_{b}^{c}=s{\bar{p}}_{c_{k}}^{v}, \end{array}$$

where *s* is the translation factor obtained by visual initialization, which has no real information.

The pure visual initialization method lacks absolute scale information. Therefore, the value estimated by the visual SFM is aligned with the IMU after preintegration to estimate the true scale. Visual-inertial alignment initialization is mainly to solve the following problems, including the initialization of gyroscope bias, the initialization of velocity, gravitational acceleration, and scale.

The first is to initialize the gyroscope. The gyroscope bias can be obtained from two consecutive key frames with known orientations, considering two consecutive frames *b*_*k*_ and *b*_*k*+1_ in the sliding window, and *q*_*bk*_^*v*^ and *q*_*b*_*k*+1__^*v*^ represent the rotations obtained from the pure visual sliding window optimization, respectively. Linearize the IMU preintegration term for the gyroscope bias and minimize the following function:

(38) $$\begin{array}{c} \min_{\delta b_{w}}\sum_{k\in ℬ}{\left\|q_{b_{k+1}}^{v-1}⊗q_{b_{k}}^{v}⊗\gamma_{b_{k+1}}^{b_{k}}\right\|}^{2}. \end{array}$$

Among them, there are:

(39) $$\begin{array}{c} \gamma_{b_{k+1}}^{b_{k}}\approx {\hat{\gamma }}_{b_{k+1}}^{b_{k}}⊗\left[J_{b_{w}}^{\gamma }\begin{array}{c} 1\\ \delta b_{w} \end{array}\right]. \end{array}$$

In formula (42), ℬ represents all the frames in the window, and the product of the two quaternions indicates that the camera rotates from the *k*th frame to the *k* + 1th frame, and the gyroscope rotates from the *k* + 1th frame to the *k*th frame, and the optimized objective function is

(40) $$\begin{array}{c} q_{b_{k+1}}^{v-1}⊗q_{b_{k}}^{v}⊗\gamma_{b_{k+1}}^{b_{k}}=\left[\begin{array}{c} 1\\ 0 \end{array}\right]. \end{array}$$

The algorithm takes $\hat{\gamma }$ into the formula and multiplies the inverse moment ordering of the relative constraints obtained from the preintegration to the left on both sides of formula (40) and obtains by Cholesky decomposition (multiplying the transpose of *J*_*b*_*w*__^*γ*^ on both sides of the formula):

(41) $$\begin{array}{c} J_{b_{w}}^{\gamma T}J_{b_{w}}^{\gamma }\delta b_{w}=2J_{b_{w}}^{\gamma T}2{\left({\hat{\gamma }}_{b_{k+1}}^{b_{k}-1}⊗q_{b_{k}}^{v}⊗q_{b_{k+1}}^{v-1}\right)}_{vec}. \end{array}$$

In this way, the initial calibration value of the gyroscope bias *b*_*w*_ can be estimated, and then the IMU preintegration terms ${\hat{\alpha }}_{b_{k+1}}^{b_{k}},{\hat{\beta }}_{b_{k+1}}^{b_{k}},{\hat{\gamma }}_{b_{k+1}}^{b_{k}}$ are corrected with the new gyroscope bias.

The second is the initialization of velocity, gravitational acceleration, and scale. The initialized state vector is as follows:

(42) $$\begin{array}{c} \chi_{I}=\left[v_{b_{0}}^{v},v_{b_{1}}^{v},\ldots v_{b_{n}}^{v},g^{v},s\right], \end{array}$$

where the state vector *v*_*b*_*k*__^*v*^ represents the speed of the visual coordinate system of the *k*th frame image, **g**^*v*^ represents the gravity vector in the visual coordinate system, and *s* is the estimated scale parameter. To sum up, the dimension of *χ*_*I*_ is 3(*n* + 1) + 3 + 1. The constraint relationship between the scale parameter and the speed of the visual SFM is as follows:

(43) $$\begin{array}{c} {\hat{z}}_{b_{k+1}}^{b_{k}}=\left[\begin{array}{c} {\hat{\alpha }}_{b_{k+1}}^{b_{k}}\\ {\hat{\beta }}_{b_{k+1}}^{b_{k}} \end{array}\right]=H_{b_{k+1}}^{b_{k}}\chi_{I}+n_{b_{k+1}}^{b_{k}}\approx ,\\ \left[\begin{array}{cccc} -q_{v}^{b_{k}}\Delta t_{k} & 0 & \frac{1}{2}q_{v}^{b_{k}}\Delta t^{2} & q_{v}^{b_{k}}\left({\bar{p}}_{b_{k+1}}^{v}-{\bar{p}}_{b_{k}}^{v}\right)\\ -q_{v}^{b_{k}} & q_{v}^{b_{k}} & q_{v}^{b_{k}}\Delta t_{k} & 0 \end{array}\right]\left[\begin{array}{c} v_{b_{k}}^{v}\\ v_{v_{k+1}}^{g^{v}}\\ g^{v} \end{array}\right], \end{array}$$

where *q*_*b*_*k*__^*V*^, *q*_*b*_*k*__^*V*^, *q*_*b*_*k*__^*V*^ are all obtained from visual SFM. *q*_*V*_^*b*_*k*_^ and *q*_*b*_*k*__^*V*^ are mutually inverse matrices. The following linear least squares problem is constructed to complete the initialization of velocity, gravitational acceleration, and scale:

(44) $$\begin{array}{c} \min_{\chi_{I}}\sum_{k\in ℬ}{\left\|{\hat{z}}_{b_{k+1}}^{b_{k}}-H_{b_{k+1}}^{b_{k}}\chi_{I}\right\|}^{2}. \end{array}$$

### 2.6. Monocular Visual Inertial Coupling Nonlinear Optimization

When coupling the visual constraint value and the IMU constraint value, the data of the inertial sensor should be introduced first, and the constraint value of the IMU on the state should be added to the optimized state vector. Then, nonlinear optimization is performed within a sliding window, and all state vectors of the sliding window are as follows:

(45) $$\begin{array}{c} \chi =\left[x_{0},x_{1},\ldots x_{n},x_{c}^{b},\lambda_{0},\lambda_{1},\ldots \lambda_{m}\right],\\ x_{k}=\left[p_{b_{k}}^{w},v_{b_{k}}^{w},q_{b_{k}}^{w},b_{a},b_{g}\right],k\epsilon \left[0,n\right],\\ x_{c}^{b}=\left[p_{c}^{b},q_{c}^{b}\right], \end{array}$$

where **x**_*k*_ represents the state of the IMU when the *k*th image is captured. There are *n* + 1 states in the sliding window, and each state contains the position, velocity, and rotation in the world coordinate system, and the IMU offsets in the IMU coordinate system. *λ*_*m*_ represents the inverse depth information of the *m*th 3D point, and **x**_*c*_^*b*^ represents the external parameter from the camera to the IMU. The objective function is

(46) $$\begin{array}{c} \min_{\chi }\left\{{\left\|r_{p}-H_{p}\chi \right\|}^{2}+\sum_{k\in ℬ}{\left\|r_{ℬ}\left({\hat{z}}_{b_{k+1}}^{b_{k}},\chi \right)\right\|}_{p_{b_{k+1}}^{b_{k}}}^{2}+\sum_{\left(l,j\right)\text{ó}C}\rho \left({\left\|r_{C}\left({\hat{z}}_{l}^{c_{j}}\right)\right\|}_{p_{l}}^{2}c_{j}\right)\right\}, \end{array}$$

where **H**_*p*_ is the Huber norm, which is defined as follows:

(47) $$\begin{array}{c} \rho \left(s\right)=\left\{\begin{array}{c} 1,s\geq 1,\\ 2\sqrt{s}-1,s<1. \end{array}\right. \end{array}$$

In formula (52), ‖·‖_*P*_ represents the Mahalanobis distance weighted by the covariance matrix **P**, and $\left\{r_{p},H_{p}\right\},r_{B}\left({\hat{z}}_{b_{k+1}}^{b_{k}},X\right)$, and $r_{C}\left({\hat{z}}_{l}^{c},X\right)$ represent the marginalized prior information, the IMU measurement residual, and the visual reprojection error, respectively. ℬ is the set of all IMU measurement frames, and *𝒞* is the set of visual features in the sliding window.

According to the Gauss–Newton method, the incremental method can be used to calculate the minimum value of the Gaussian objective function, as follows:

(48) $$\begin{array}{c} \min_{\delta X}{\left\|r_{ℬ}\left({\hat{z}}_{b_{k+1}}^{b_{k}},\chi +\Delta X\right)\right\|}_{p_{b_{k+1}}^{b_{k}}}^{2}=\min_{\delta X}{\left\|r_{ℬ}\left({\hat{z}}_{b_{k+1}}^{b_{k}},\chi \right)+J_{b_{k+1}}^{b_{k}}\Delta X\right\|}_{p_{b_{k+1}}^{b_{k}}}^{2}, \end{array}$$

where *J*_*b*_*k*+1__^*b*_*k*_^ is the Jacobian matrix of the error term *r*_ℬ_ with respect to all state vectors *χ*.

The algorithm differentiates the above formula from Δ*X* and then sets its derivative to 0, and the formula for the increment Δ*X* can be calculated as follows:

(49) $$\begin{array}{c} J_{b_{k+1}}^{b_{k}T}P_{b_{k+1}}^{b_{k}-1}J_{b_{k+1}}^{b_{k}}\Delta X=-J_{b_{k+1}}^{b_{k}T}P_{b_{k+1}}^{b_{k}-1}J_{b_{k+1}}^{b_{k}}r_{ℬ}. \end{array}$$

The overall incremental equation of the objective function is as follows:

(50) $$\begin{array}{c} \left(H_{p}+\sum J_{b_{k+1}}^{b_{k}T}P_{b_{k+1}}^{b_{k}-1}J_{b_{k+1}}^{b_{k}}+\sum J_{l}^{c_{j}^{T}}P_{l}^{c_{j}-1}J_{l}^{c_{j}}\right)\Delta X=b_{p}+\sum J_{b_{k+1}}^{b_{k}T}P_{b_{k+1}}^{b_{k}-1}r_{ℬ}+\sum J_{l}^{C_{j}^{T}}P_{l}^{C_{j}^{-1}}r_{C}, \end{array}$$

where *P*_*b*_*k*+1__^*b*_*k*_^ represents the covariance of the IMU preintegrated noise term and *P*_*l*_^*C*_*j*_^ represents the visually observed noise covariance. When the noise covariance *P*_*b*_*k*+1__^*b*_*k*_^ of the IMU is larger, the inverse of *P*_*b*_*k*+1__^*b*_*k*_^, that is, its information matrix is smaller, which means that the IMU observations are not as reliable as the visual observations. Formula (50) can be simplified to

(51) $$\begin{array}{c} \left(\Lambda_{p}+\Lambda_{B}+\Lambda_{C}\right)\Delta X=b_{p}+b_{B}+b_{C}, \end{array}$$

where Λ_*p*_, Λ_*B*_, and Λ_*C*_ represent the Hessian matrix. Using the perturbation method to calculate, we can get

(52) $$\begin{array}{c} J=\frac{\partial r}{\partial X}\\ =\underset{\delta X⟶0}{\lim}\frac{r\left(X⊕\delta X\right)-r\left(X\right)}{\delta X}, \end{array}$$

where *δX* represents the small disturbance of the state vector instead of the incremental Δ*X*, ⊕ represents the disturbance of the state vector.

The continuous preintegration formula is derived in the IMU preintegration, and the IMU residuals are as follows:

(53) $$\begin{array}{c} r_{ℬ}\left({\hat{z}}_{b_{k+1}}^{b_{k}},\chi \right)=\left[\begin{array}{c} \delta \alpha_{b_{k+1}}^{b_{k}}\\ \delta \beta_{b_{k+1}}^{b_{k}}\\ \delta \theta_{b_{k+1}}^{b_{k}}\\ \delta b_{a}\\ \delta b_{g} \end{array}\right]\\ =\left[\begin{array}{c} R_{w}^{b_{k}}\left(p_{b_{k+1}}^{w}-p_{b_{k}}^{w}-v_{b_{k}}^{w}\Delta t_{k}+\frac{1}{2}g^{w}\Delta t_{k}^{2}\right)-\alpha_{b_{k+1}}^{b_{k}}\\ R_{w}^{b_{k}}\left(v_{b_{k+1}}^{w}\Delta t_{k}-v_{b_{k}}^{w}\Delta t_{k}+g^{w}\Delta t_{k}\right)-\beta_{b_{k+1}}^{b_{k}}\\ 2{\left[\gamma_{b_{k+1}}^{b_{k}}⊗q_{b_{k}}^{w}⊗q_{b_{k+1}}^{w}\right]}_{xyz}\\ b_{a_{b_{k+1}}}-b_{a_{b_{k}}}\\ b_{\omega_{b_{k+1}}}-b_{\omega_{b_{k}}} \end{array}\right]. \end{array}$$

According to the above formula, it can be seen that the optimization variables of the IMU residual mainly include the position, rotation, speed, and inertia bias at the *i* and *j* times:

(54) $$\begin{array}{c} \left[p_{b_{k}}^{w},q_{b_{k}}^{w}\right],\left[v_{b_{k}}^{w},b_{a_{k}},b_{\omega_{k}}\right],\left[p_{b_{k+1}}^{w},q_{b_{k+1}}^{w}\right],\left[v_{b_{k+1}}^{w},b_{a_{k+1}},b_{\omega_{k+1}}\right]. \end{array}$$

To calculate the Jacobian matrix, perturbation is added to each optimization variable to obtain

(55) $$\begin{array}{c} \left[\delta p_{b_{k}}^{w},\delta \theta_{b_{k}}^{w}\right],\left[\delta v_{b_{k}}^{w},\delta b_{a_{k}},\delta b_{\omega_{k}}\right],\left[\delta p_{b_{k+1}}^{w},\delta \theta_{b_{k+1}}^{w}\right],\left[\delta v_{b_{k+1}}^{w},\delta b_{a_{k+1}},\delta b_{\omega_{k+1}}\right]. \end{array}$$

Taking the partial derivatives for the above disturbance variables, respectively, we can get

(56) $$\begin{array}{c} J{\left[0\right]}^{15\times 7}=\left[\frac{\partial r_{B}}{\delta p_{b_{k}}^{w}},\frac{\partial r_{B}}{\delta \theta_{b_{k}}^{w}}\right],\\ J{\left[1\right]}^{15\times 7}=\left[\frac{\partial r_{B}}{\delta v_{b_{k}}^{w}},\frac{\partial r_{B}}{\delta b_{a_{k}}},\frac{\partial r_{B}}{\delta b_{\omega_{k}}}\right],\\ J{\left[2\right]}^{15\times 7}=\left[\frac{\partial r_{B}}{\delta p_{b_{k+1}}^{w}},\frac{\partial r_{B}}{\delta \theta_{b_{k+1}}^{w}}\right],\\ J{\left[3\right]}^{15\times 7}=\left[\frac{\partial r_{B}}{\delta v_{b_{k+1}}^{W}},\frac{\partial r_{B}}{\delta b_{a_{k+1}}},\frac{\partial r_{B}}{\delta b_{\omega_{k+1}}}\right]. \end{array}$$

**Figure 9:**
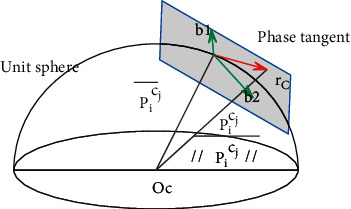
Unit spherical projection model.

The visual residual is a reprojection error, which represents the difference between the estimated value and the observed value of the feature point in the normalized camera coordinate system. The small receiver camera used in this paper belongs to the fisheye camera model and belongs to the fisheye with a large viewing angle, so its projection on the unit sphere needs to be considered, as shown in Figure 9.

Through the unit spherical projection model illustrated in the figure above, the value of the visual residual is decomposed into two directions. The final visual residual model looks like this:

(57) $$\begin{array}{c} r_{c}\left({\hat{z}}_{l}^{c_{j}},\chi \right)={\left[b_{1},b_{2}\right]}^{T}\cdot \left({\hat{p}}_{l}^{c_{j}}-\frac{p_{l}^{c_{j}}}{\left\|p_{l}^{c_{j}}\right\|}\right),\\ {\hat{p}}_{l}^{c_{j}}=\pi_{c}^{-1}\left(\left[\begin{array}{c} {\hat{u}}_{l}^{c_{j}}\\ {\hat{v}}_{l}^{c_{j}} \end{array}\right]\right),\\ p_{l}^{c_{j}}=R_{c}^{b}\left\{R_{w}^{b_{j}}\left[R_{b_{i}}^{w}\left(R_{c}^{b}\frac{1}{\lambda_{l}}{\bar{P}}_{l}^{c_{i}}+p_{c}^{b}\right)+p_{b_{i}}^{w}-p_{b_{j}}^{w}\right]-p_{c}^{b}\right\}, \end{array}$$

where ${\hat{\bar{p}}}_{l}^{c_{j}}$ and *p*_*l*_^*c*_*j*_^ represent the estimated and observed coordinates of the landmark point 1 in the *j*-th frame image under the normalized camera coordinate system, respectively. The optimization variables of the visual residual are as follows:

(58) $$\begin{array}{c} \left[p_{b_{i}}^{w},q_{b_{i}}^{w}\right],\left[p_{b_{j}}^{w},q_{b_{j}}^{w}\right],\left[p_{c}^{b},q_{c}^{b}\right],\lambda_{l}, \end{array}$$

where *λ*_*l*_ represents the inverse depth value when the landmark point 1 is first observed by the *j*-th image. The inverse depth is used as the optimization variable because the inverse depth satisfies the Gaussian distribution, and it can reduce the parameter variables in the actual optimization process. According to the above formula, by adding disturbance to each optimization variable, the following Jacobian is obtained:

(59) $$\begin{array}{c} J{\left[0\right]}^{3\times 7}=\left[\frac{\partial r_{C}}{\partial p_{b_{i}}^{w}},\frac{\partial r_{C}}{\partial q_{b_{i}}^{w}}\right],\\ J{\left[2\right]}^{3\times 7}=\left[\frac{\partial r_{C}}{\partial p_{c}^{b}},\frac{\partial r_{C}}{\partial q_{c}^{b}}\right], \end{array}$$

(60) $$\begin{array}{c} J{\left[3\right]}^{3\times 1}=\frac{\partial r_{C}}{\partial \lambda_{l}}. \end{array}$$

## 3. E-Sports Training System Based on Intelligent Gesture Recognition

**Figure 10:**
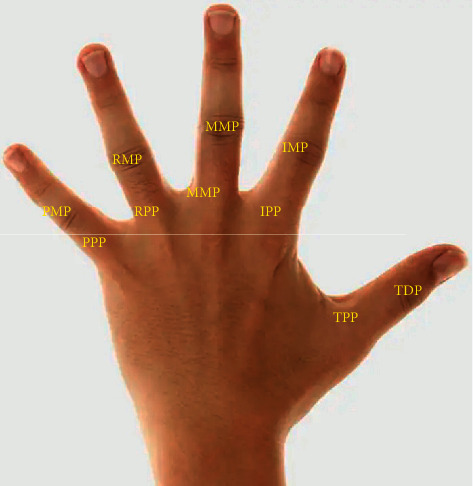
Demarcation boundaries of hand movements.

This paper combines the finger joints and the sensor in the data glove to demarcate the movement of the finger joints. This paper mainly considers the distal phalanx of the thumb (TDP) and the proximal joint proximal phalanx of the thumb (TPP) of the thumb as shown in Figure 10 and the changes in the intermediate joints middle phalanges (MP) and proximal phalanges (PP) of the remaining four fingers.

**Figure 11:**
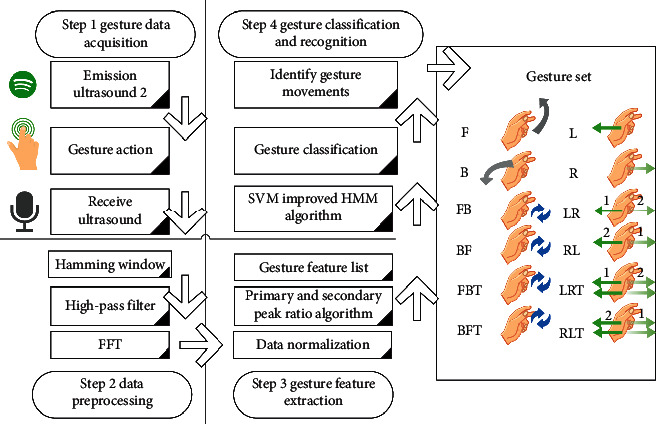
Overall system framework.

This paper combines the algorithm part of the second part to construct the e-sports training system, and the overall framework of the system is shown in Figure 11.

The simulation of the system proposed in this paper is carried out through the MATLAB platform, and the gesture recognition effect of the system and the application effect in the e-sports training system are evaluated, and the obtained results are shown in Tables 1 and 2.

It can be seen from the above research that the electronic economic training system based on intelligent gesture recognition proposed in this paper has certain effects.

## 4. Conclusion

As emerging sports, e-sports are mainly participated by the younger generation, which has the characteristics of younger and younger age. E-sports can exercise people's thinking ability, psychological pressure resistance, unity and cooperation, hand-eye coordination, and so on. Moreover, in the process of participating in e-sports, it can also make the younger generation have the awareness of abiding by the rules, cultivate the participants to have a fair and open, never admit defeat, pursue a stronger competitive spirit, and have a positive impact on the participants' lives. This paper combines the intelligent gesture recognition technology and the construction of the performance e-sports training system and judges the training effect of the players through the player gesture recognition. The research shows that the electronic economic training system based on intelligent gesture recognition proposed in this paper has certain effects.

## Figures and Tables

**Table 1:** Gesture recognition effect of the system.

Number	Gesture recognition	Number	Gesture recognition	Number	Gesture recognition
1	86.095	13	87.363	25	86.633
2	88.782	14	89.124	26	90.414
3	90.695	15	90.055	27	86.697
4	86.325	16	89.968	28	86.916
5	86.705	17	88.482	29	89.003
6	89.557	18	91.278	30	88.446
7	86.623	19	89.215	31	89.911
8	91.281	20	86.272	32	90.202
9	88.050	21	91.253	33	89.507
10	87.621	22	86.770	34	88.130
11	88.498	23	86.843	35	91.326
12	86.304	24	90.614	36	86.846

**Table 2:** The application effect of the method proposed in this paper in the e-sports training system.

Number	Training effect	Number	Training effect	Number	Training effect
1	82.685	13	82.234	25	79.365
2	80.315	14	78.537	26	81.356
3	79.700	15	81.913	27	78.790
4	78.179	16	82.938	28	79.700
5	82.942	17	78.167	29	78.194
6	80.821	18	78.250	30	78.253
7	81.176	19	81.512	31	80.133
8	78.183	20	80.076	32	82.668
9	82.636	21	81.925	33	83.747
10	81.613	22	82.857	34	82.354
11	80.514	23	82.951	35	78.416
12	80.913	24	79.066	36	83.128

## Data Availability

The labeled data set used to support the findings of this study is available from the corresponding author upon request.
